# Calcium ionophore improves embryonic development and pregnancy outcomes in patients with previous developmental problems in ICSI cycles

**DOI:** 10.1186/s12884-022-05228-3

**Published:** 2022-12-02

**Authors:** Xiaolei Chen, Haibin Zhao, Jiale Lv, Yi Dong, Maoning Zhao, Xinlei Sui, Ran Cui, Boyang Liu, Keliang Wu

**Affiliations:** 1 Center for Reproductive Medicine, Cheeloo College of Medicine, Shandong University, Jinan, 250012 Shandong China; 2 Key Laboratory of Reproductive Endocrinology of Ministry of Education, Shandong University, Jinan, 250012 Shandong China; 3 Shandong Key Laboratory of Reproductive Medicine, Jinan, 250012 Shandong China; 4 Shandong Provincial Clinical Research Center for Reproductive Health, Jinan, 250012 Shandong China; 5 Shandong Technology Innovation Center for Reproductive Health, Jinan, 250012 Shandong China; 6 National Research Center for Assisted Reproductive Technology and Reproductive Genetics, Shandong University, Jinan, 250012 Shandong China

**Keywords:** Poor embryo development, Assisted oocyte activation, Calcium ionophore, Age

## Abstract

**Background:**

Calcium (Ca^2+^) ionophores are now mainly considered as efficient treatments for fertilization failure. Recently, its application for rescuing poor embryo development was proposed but still non-routine. This study aimed to explore whether Ca^2+^ ionophore improves embryo development and pregnancy outcomes in patients with poor embryo development in previous intracytoplasmic sperm injection (ICSI) cycles.

**Methods:**

This study included 97 patients undergoing assisted oocyte activation (AOA) with Ca^2+^ ionophore (calcimycin, A23187) treatment. Preimplantation embryonic development and clinical outcomes were compared between ICSI-AOA cycles (AOA group) and previous ICSI cycles of the same patients in which poor embryo developmental potential was present (non-AOA group). Subgroups stratified by maternal age (< 35, 35–40, ≥ 40 years, respectively) were analyzed separately.

**Results:**

A total of 642 MII oocytes were collected in AOA group, and 689 in non-AOA group. Significantly higher day 3 good quality embryo rate (*P* = 0.034), good quality blastocyst formation rate (*P* <  0.001), and utilization rate (P <  0.001) were seen in AOA group. Similar results were seen in each subgroup. For pregnancy outcomes, there were significant differences in clinical pregnancy rate (*P* = 0.039) and live birth rate (*P* = 0.045) in total group. In subgroup aged < 35 years, biochemical (*P* = 0.038), clinical (*P* = 0.041), and ongoing pregnancy rate (*P* = 0.037) in AOA group were significantly higher than that in non-AOA group. No significant improvement for clinical outcomes for subgroups aged 35–40 and aged ≥40.

**Conclusion:**

The study suggests that calcimycin could improve preimplantation development and pregnancy outcomes in patients aged < 35 years with embryo developmental problems in previous ICSI cycles.

**Supplementary Information:**

The online version contains supplementary material available at 10.1186/s12884-022-05228-3.

## Introduction

Infertility has become a health problem of global concern affecting about 15% of couples and 80% of them could find a solution with the advancement of assisted reproductive technology (ART) [1]. Part of failed patients undergoing in vitro fertilization (IVF) suffered from poor embryo developmental potential. Embryo quality positively correlated to pregnancy outcomes [2, 3], but the cause of poor embryo development is still unclear and there is a lack of effective treatment in clinic [4, 5].

Some studies reported that Calcium (Ca^2+^) signal deficiency or insufficiency during oocyte activation is associated with embryo arrest, cleavage anomalies, or poor preimplantation development and blastocyst quality [6–9]. During mammalian fertilization, sperm releases sperm-specific phospholipase C zeta (PLCζ) into the ooplasm immediately after the fusion of sperm and oocyte plasma membranes, and then results in Ca^2+^ oscillations which are essential for normal fertilization and the onset of embryogenesis by triggering downstream signaling pathways. This process is known as oocyte activation [10–12]. A sequence of cellular events during oocyte activation, including maternal mRNA recruitment, cytoskeletal rearrangement, embryonic genome activation, and initiation of cleavage [13–16], is the premise of oocyte-to-embryo transition and early embryonic development [11, 17, 18] and dependents on various amount of Ca^2+^ oscillations [13]. In general, increase of Ca^2+^ during activation has later effect on subsequent embryonic development [19, 20] and oppositely, oocyte activation failure resulting from sperm- or oocyte-related calcium insufficiency could lead to poor embryo development.

Assisted oocyte activation (AOA), including mechanical, electrical, and chemical stimuli, has been considered as a feasible approach which can increase Ca^2+^ in the oocyte and induce oocyte activation efficiently [8]. Calcium ionophores including ionomycin and calcimycin (A23187) are the most common approaches used for AOA in ART [21], and have been widely used for rescuing fertilization failure and globozoospermia [22]. Recently, researchers tried to improve embryo development with AOA at intracytoplasmic sperm injection (ICSI) in patients with various early embryo developmental problems. It has been reported that successful cleavage after calcium ionophore treatment could be achieved in cases with previous embryo arrest at the pronuclear stage [23]. A multicentric prospective study reported that calcium ionophore could rescue patients with completely developmental arrest or delay, or reduced blastocyst formation rate [24]. But a study using sibling oocytes as the control group in the same cycle reported that calcium ionophore was not able to improve embryo laboratory outcomes as well as pregnancy outcomes in patients who suffered from poor embryo development but achieved more than 70% normal fertilization rate in previous ICSI cycles [25].

Previous studies were still sparse, based on small sample size, and no consensus has been reached. Of central concern, AOA treatment protocols as well as targeted patients were obviously different among these studies. AOA was sometimes performed immediately after ICSI for 15 minutes and sometimes performed after 60 minutes of ICSI for 10 minutes [24–26]. In fact, Ca^2+^ oscillations begin at 15–30 minutes after ICSI [27, 28], which might be relevant to delayed release of PLCζ due to delayed disintegration of spermatozoa plasma membrane at ICSI [29]. Thus, we applied calcium ionophore at 15–30 minutes after ICSI and aimed to evaluate the effect of calcium ionophore on patients with poor embryo developmental potential in previous ICSI cycles.

## Materials and methods

### Patients

The study was performed at the Hospital for Reproductive Medicine Affiliated to Shandong University. A total of 97 female patients undergoing calcimycin (A23187) treatment for AOA (AOA group) were collected from March 2020 to December 2021 and met one of the following AOA indications in previous ICSI cycles: (1) no good quality embryo on day 3 and no good quality blastocyst; (2) at least one good quality embryo on day 3 but no good quality blastocyst; (3) or less than 20% good quality blastocyst formation rate [24]. Their preceding conventional ICSI cycles without AOA served as the control group (non-AOA group). Patients with normal fertilization rate < 30% in previous ICSI cycles were excluded because that is conventional indication for AOA [24]. Patients with MII oocyte retrieved < 2 were also ruled out, which was a compromise between minimizing the contingency of poor embryo development and adequate sample size. The patients were divided into three subgroups by their age (< 35, 35–40, ≥ 40 years, respectively) in order to explore the effect of AOA at different age stages.

### ICSI and ionophore oocyte activation

Controlled ovarian hyperstimulation (COH) protocols were determined by experienced physicians depending on the patients’ baseline data and willing. Follicle growth was initiated by either recombinant follicle stimulating hormone or human menopausal gonadotropin in general. Ovulation was induced by human chorionic gonadotropin (hCG) at a dose of 4000–10,000 IU. Oocyte retrieval was performed 36 hours after hCG administration. The time interval between removing granule cells and performing ICSI in non-AOA cycles was same as that in AOA cycles. Routinely, the time interval ranges from 0.5 h to 2 h, which depends on the maturation rate of the obtained oocytes and the number of ICSI patients on the day of oocyte retrieval. The ICSI procedure was identical in both non-AOA and AOA cycles and performed by experienced operators. Sperm used for ICSI were all fresh sperm and most were ejaculated sperm. During calcium ionophore oocyte activation cycles, MII oocytes were exposed to A23187 (5 μM, Sigma, St. Louis, MO, USA) for 15 minutes after 15 minutes of ICSI, and then rinsed for three times. The base solution for oocyte activation is cleavage-stage medium, which is G1-plus (Vitrolife) in our center.

### Embryo culture and transfer

Embryos were cultured with sequential culture media supplied by Vitrolife (G-IVF, G1 and G2; Scandinavian IVF Science, Sweden). Puissant’s criteria [30] was used for embryo scoring on day 3. Embryo transfer was considered on the third day of embryo culture when at least one available embryo was formed, and no more than three fresh cleaved embryos were transferred at once. Alternatively, all embryos were cultured up to blastocyst stage. Blastocyst scores were assessed according to Gardner and Lane’s criteria [31]. In general, only blastocysts over 4 BC grade were graded as good quality and transferrable in our center. If there were transferrable blastocysts, fresh embryo transfer with one blastocyst was performed. Surplus embryos after fresh embryo transfers were all incubated up to blastocyst stage and frozen. During IVF cycles, if there was a risk of ovarian hyperstimulation syndrome, or there were any conditions that might have impaired endometrial receptivity or patients’ specific wishes, embryos were all cultured for blastocyst cryopreservation and fresh embryo transfer would not be performed. Embryos used for frozen embryo transfers were all at blastocysts stage in the study and only one blastocyst was transferred in each frozen embryo transfer cycle. Frozen embryo transfer procedure was reported previously [32, 33].

### Outcomes

Primary outcomes included day 3 good quality embryo rate (good quality embryos on day 3/2PN (pronuclear) zygotes), and good quality blastocyst formation rate (good quality blastocysts/2PN zygotes). Secondary outcomes were utilization rate ((day 3 good quality embryos transferred*0.55 + day 3 non-good quality embryos transferred*0.45 + blastocyst transferred or frozen*1.00)/MII oocytes), biochemical pregnancy (a rising hCG concentration > 25 IU/L 12 days after transfer) rate, clinical pregnancy (visualization of gestational sacs through an abdominal ultrasound) rate, miscarriage (spontaneous pregnancy loss before 28 weeks of gestation) rate, and live birth (delivering at least one live-born infant after ≥28 weeks of gestation) rate.

### Statistical analysis

Variables were tested for normal distribution using Kolmogorov-Smirnov test or Shapiro-Wilk test based on the sample size. According to the outcome of distribution, either paired t test or Wilcoxon sign rank test was applied for descriptive data and McNemar test or chi-square test for categorical variables.

All statistical analyses were performed using SPSS software, version 26.0. A 2-sided *P* value less than 0.05 was considered statistically significant.

## Results

### Participants

A total of 97 female patients were included in this study and each patient was included only once. Patients underwent ICSI were due to unexplained infertility (44.33%), male factor infertility (41.24%), and female factor infertility (14.43%). The mean (± SD) maternal age was 34.84 ± 4.92 years, body mass index (BMI) was 23.87 ± 2.86 kg/m^2^, anti-Mullerian hormone (AMH) was 2.91 ± 2.20 ng/ml, and they had undergone 2.24 ± 1.40 previous ICSI cycles. No significant difference was observed between non-AOA and AOA cycles in total group and each subgroup as far as COH protocols and gonadotropin dose were concerned (Table 1 and Table S1 in Additional File 1). There was no significant difference between non-AOA and AOA cycles in terms of growth hormone application in each subgroup (Table S2 in Additional File 1). 7 male patients supplied epididymal or testicular sperm in their both AOA cycles and non-AOA cycles and the rest were all ejaculated sperm (Table S2 in Additional File 1).

**Table 1:** COH characteristics and preimplantation development outcomes in non-AOA and AOA group for total patients

	Non-AOA	AOA	*P* value
Cycles, n	97	97	
Age, year	33.62 ± 5.25	34.84 ± 4.92	
COH protocol, n (%)			0.122
GnRH-agonist protocol	50 (51.55%)	46 (47.42%)	
GnRH-antagonist protocol	38 (39.18%)	36 (37.11%)	
Other unconventional protocol	9 (9.28%)	15 (15.46%)	
Gn total dose, IU	2171.55 ± 932.35	2273.33 ± 1018.15	0.379
No. of MII oocytes, n	689	642	
Normal fertilization rate, n (%)	503 (73.00%)	422 (65.73%)	0.006*
Multi-PN formation rate, n (%)	19 (2.76%)	22 (3.43%)	0.112
Cleavage rate, n (%)	493 (98.01%)	413 (97.87%)	0.575
Day 3 good quality embryo rate, n (%)	145 (28.83%)	163 (38.63%)	0.034*
Good quality blastocyst formation rate, n (%)	18 (3.58%)	117 (27.73%)	< 0.001*
Utilization rate, n (%)	50.60 (7.34%)	144.65 (22.53%)	< 0.001*

Values are presented as mean ± SD or n (%). ^*^P < 0.05.

COH, controlled ovarian hyperstimulation; Gn, gonadotropin; MII, metaphase II; PN, pronuclear.

### Total included patients

When all included infertile patients were analyzed, 642 MII oocytes for ICSI were collected in AOA group and 689 in non-AOA group. After ICSI, significantly lower normal fertilization rate was found in AOA group (*P* = 0.006). There was no significant difference in multi-PN formation rate (*P* = 0.112) and cleavage rate (*P* = 0.575). However, day 3 good quality embryo rate (*P* = 0.034), good quality blastocyst formation rate (*P* < 0.001), and utilization rate (*P* < 0.001) were significantly higher in AOA group than that in non-AOA group (Table 1).

Seventy-one embryos were transferred in 46 transfer cycles in non-AOA group and 86 embryos were transferred in 69 transfer cycles in AOA group. No significant difference in terms of biochemical pregnancy rate (*P* = 0.098), but clinical pregnancy rate (*P* = 0.039), ongoing pregnancy rate (*P* = 0.021), and live birth rate (*P* = 0.045) were significantly higher in AOA group than that in non-AOA group. 4 babies were delivered in non-AOA group and 16 in AOA group. No significant difference in early miscarriage rate (*P* = 0.726) and late miscarriage rate (*P* = 0.421) between AOA group and non-AOA group (Table 2). One ectopic pregnancy occurred in non-AOA group. No birth defects were found.

**Table 2:** Clinical outcomes in non-AOA and AOA group for total patients

	Non-AOA	AOA	*P* value
Embryo transfer cycles, n	46	69	
No. of embryos transferred, n	71	86	
Biochemical pregnancy rate, %	28.26% (13/46)	43.48% (30/69)	0.098
Clinical pregnancy rate, %	19.57% (9/46)	37.68% (26/69)	0.039*
Ongoing pregnancy rate, %	10.87% (5/46)	28.99% (20/69)	0.021*
Ectopic pregnancy rate, %	11.11% (1/9)	0	–
Pregnancy loss rate, %			
Early miscarriage rate	37.50% (3/8)	23.08% (6/26)	0.726
Late miscarriage rate	12.50% (1/8)	3.85% (1/26)	0.421
Live birth rate, %	8.70% (4/46)	23.19% (16/69)	0.045*

Values are presented as % (n/total n). ^*^P < 0.05

Significantly higher proportion of cryo-embryo transfer (*P* < 0.001) and blastocyst transfer (*P* < 0.001) were noted in AOA group (Table S3 in Additional File 1). When pregnancy outcomes of cleavage and blastocyst transfers were analyzed separately, there was no significant difference between AOA and non-AOA group in cleavage-stage embryo transfer cycles in terms of biochemical, clinical and ongoing pregnancy rate as well as live birth rate (*P* = 0.441, *P* = 0.645, *P* = 0.109, *P* = 0.722, *P* = 0.722, respectively) (Table 3). In blastocyst transfer cycles, quality of embryos transferred in AOA group is higher than that in non-AOA group although the difference was not statistically significant (Table S4 in Additional File 1). Significantly higher biochemical pregnancy rate (*P* = 0.003) and clinical pregnancy rate (*P* = 0.008) were observed in AOA group than that in non-AOA group, while no significant difference in ongoing pregnancy rate (*P* = 0.092) was found in spite of a discrepancy of nearly 20%. No live birth was achieved in non-AOA group and 12 babies were born in AOA group (0 vs. 27.91%). 3 ongoing pregnancies in AOA group were still in the third trimester (Table 3).

**Table 3:** Pregnancy outcomes in non-AOA and AOA group for cleavage-stage embryo transfer and blastocyst transfer cycles

	Cleavage-stage embryo transfer			Blastocyst transfer		
	Non-AOA	AOA	*P* value	Non-AOA	AOA	*P* value
Biochemical pregnancy rate, %	36.36% (12/33)	26.92% (7/26)	0.441	7.69% (1/13)	53.49% (23/43)	0.003*
Clinical pregnancy rate, %	24.24% (8/33)	19.23% (5/26)	0.645	7.69% (1/13)	48.84% (21/43)	0.008*
Ongoing pregnancy rate, %	12.12% (4/33)	15.38% (4/26)	0.722	7.69% (1/13)	37.21% (16/43)	0.092
Live birth rate, %	12.12% (4/33)	15.38% (4/26)	0.722	0	27.91% (12/43)	–

Values are presented as % (n/total n). *P < 0.05

### Subgroup analysis

Fifty-two patients were included in the subgroup aged < 35 years, 27 in the subgroup aged 35–40 years, and 18 in the subgroup aged ≥40 years. Preimplantation embryonic development outcomes for each subgroup are shown in Table S1 (Table S1 in Additional File 1).

In women aged < 35 years, 408 MII oocytes were collected in non-AOA group and 399 in AOA group. Normal fertilization rate, multi-PN formation rate and cleavage rate did not differ between AOA and non-AOA group (*P* = 0.063, *P* = 0.701, *P* = 0.735, respectively). However, AOA significantly improved day 3 good quality embryo rate (*P* = 0.001), good quality blastocyst formation rate (*P* < 0.001), and utilization rate (*P* < 0.001).

In women aged 35–40, 175 MII oocytes were retrieved in non-AOA group and 148 in AOA group. There were no significant differences in normal fertilization rate, multi-PN formation rate, cleavage rate, and day 3 good quality embryo rate between AOA group and non-AOA group (*P* = 0.669, *P* = 0.285, *P* = 0.109, *P* = 0.540, respectively). Good quality blastocyst formation rate (*P* < 0.001) and utilization rate (*P* = 0.001) were significantly higher in AOA group than that in non-AOA group.

In women aged ≥40 years, 106 MII oocytes were obtained in non-AOA group and 95 in AOA group. Normal fertilization rate was significantly lower in AOA group (*P* = 0.007). There were 3 multi-PN zygotes in AOA group and no multi-PN zygotes were formed in non-AOA group (3.16% vs. 0). No significant difference in cleavage rate and day 3 good quality embryo rate between AOA and non-AOA group was found (*P* > 0.999, *P* = 0.789, respectively). Nevertheless, AOA improved good quality blastocyst formation rate (*P* = 0.002) and utilization rate (*P* = 0.006) significantly.

**Fig. 1:**
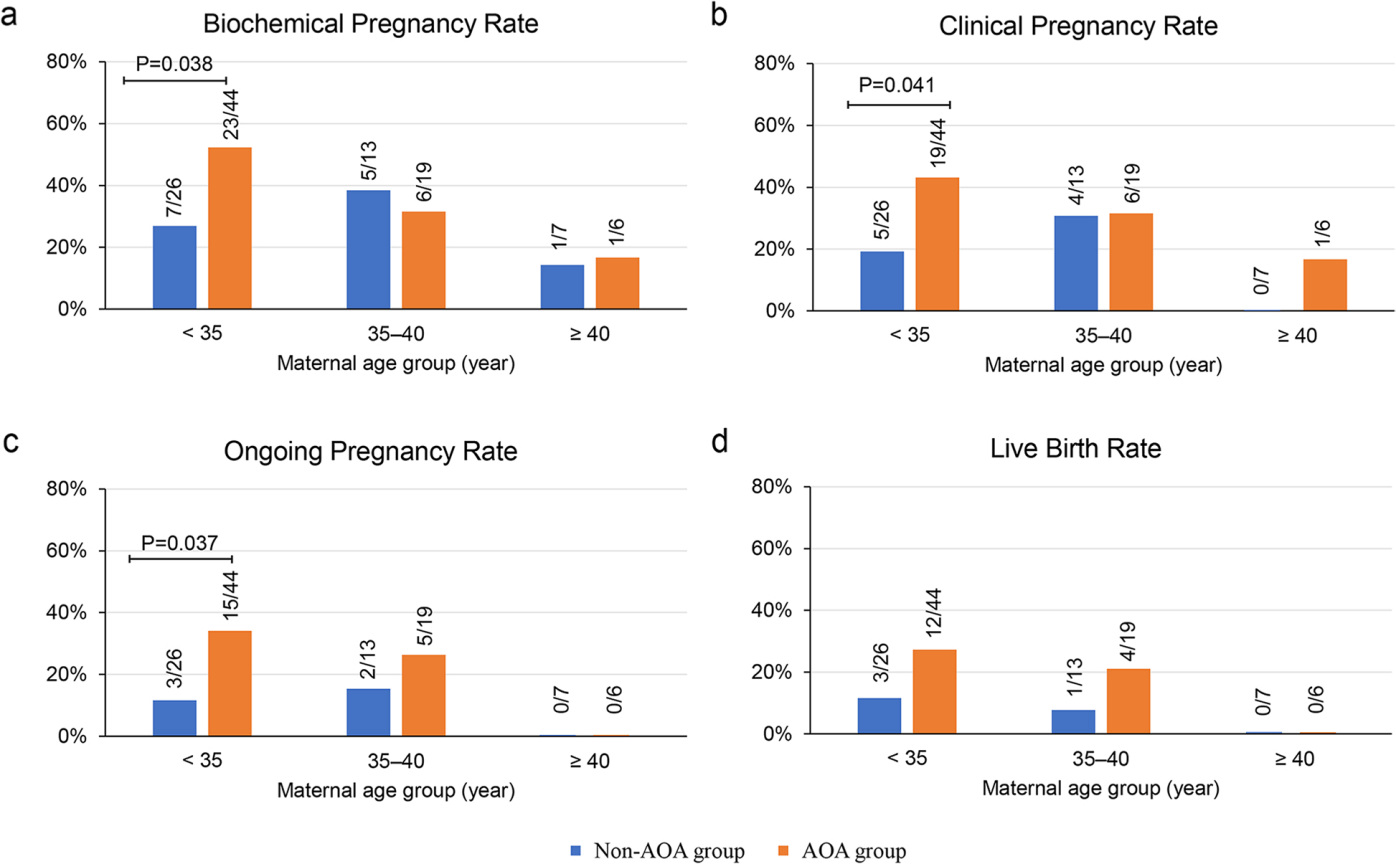
Comparison of clinical outcomes between non-AOA and AOA group for each subgroup. **a** biochemical pregnancy rate, **b** clinical pregnancy rate, **c** ongoing pregnancy rate, **d** live birth rate. Fractions above bars indicate the proportion of embryo transfers that achieved biochemical pregnancy, clinical pregnancy, ongoing pregnancy or live birth

In terms of pregnancy outcomes, significant difference between non-AOA group and AOA group was only observed in subgroup aged < 35 years. For this subgroup, 42 embryos were transferred in 26 transfer cycles in non-AOA group and 56 embryos were transferred in 44 transfer cycles in AOA group. More cryo-embryo transfers (*P* = 0.004) and blastocyst transfers (*P* = 0.005) were carried out in AOA group (Table S3 in Additional File 1). Biochemical pregnancy rate (*P* = 0.038), clinical pregnancy rate (*P* = 0.041), and ongoing pregnancy rate (*P* = 0.037) were statistically higher in AOA group compared with non-AOA group. 3 babies were delivered in non-AOA group and 12 babies in AOA group. No significant difference in live birth rate between non-AOA and AOA group was seen (11.54% vs. 27.27%, *P* = 0.121) (Fig. 1). Of note, 3 ongoing pregnancies in AOA cycles (33 weeks, 35 weeks, 39 weeks of gestation, respectively) had not completed clinical follow-up yet when the article was done. There was one early pregnancy loss in non-AOA group and 4 in AOA group, which was not significantly different (*P* > 0.999) (Table S5 in Additional File 1). In subgroups aged 35–40 and aged ≥40, no significant difference between non-AOA group and AOA group was observed in terms of biochemical pregnancy rate, clinical pregnancy rate, ongoing pregnancy rate, live birth rate (Fig. 1), as well as pregnancy loss rate (Table S5 in Additional File 1).

## Discussion

Calcium ionophore can promote extracellular calcium influx as well as calcium release from intracellular store, which can compensate oocyte- or sperm-borne calcium insufficiency [34]. Improvements in cases of globozoospermia or fertilization failure with the application of calcium ionophores for AOA have been repeatedly demonstrated [22, 35]. However, only few studies explored its effect in cases with embryonic development problems and no consistent conclusions were achieved yet. Clinical use of calcium ionophore in these patients is still not considered as conventional treatment measure. Given the importance of calcium for oocyte activation and embryonic development, we supposed that Ca^2+^ oscillations induced by PLCζ after sperm-ooplasm fusion were far from enough to complete preimplantation embryonic development in patients with previous ICSI failure due to embryo developmental problems. Once intracellular calcium stores have been exhausted, oocytes need exogenous calcium supplement [36].

In this study, we showed that calcimycin treatment significantly improved day 3 good quality embryo rate, good blastocyst formation rate, as well as utilization rate. Calcimycin seemed be able to help embryos step over the developmental arrest point by making up for calcium deficiency. Moreover, advanced age does not limit the application of calcimycin. Meanwhile, calcimycin significantly improved post-implantation development as well as pregnancy outcomes. Importantly, significant improvement of clinical outcomes only observed in women < 35 years old. Higher implantation rate after AOA was reported as long as the oocytes were derived from patients younger than 36 years [37]. It is well known that fertility declines with maternal age because of reduced oocyte quality except for premature follicles recruitment, increasing ovulatory disorders, and impaired luteal phase [38]. Intrinsic defect of oocytes from patients with advanced age, such as oocyte aneuploidy and mitochondrial dysfunction, instead of oocyte activation failure, is responsible to age-related infertility and may not be able to be rescued by AOA [26]. Besides, current ionophores used in human oocytes can only induce single transient Ca^2+^ shock, unlike Ca^2+^ oscillations in physiologic condition, but their benefits were still significant [21]. Calcium ionophore may activate fully cellular events during oocyte activation which are essential for embryo development by promoting adequate calcium release for oocyte activation. Multiple lines of evidence showed that oocyte activation and subsequent development were results of calcium signal summation [39], and oocytes can be fully activated as long as calcium release reaches a specific threshold [13]. And the molecular effects induced by ionophores are comparable with that in physiologic condition [17]. Oocytes can be tolerant to the change of physiologic calcium pattern to some extent [36]. However, single Ca^2+^ transient induced by AOA is still considered as a limited solution. We suspected that oocytes derived from women of advanced age cannot be tolerant to artificial Ca^2+^ fluctuations as the younger women. Excessive Ca^2+^ after AOA may be even noxious stimuli for aging oocytes.

Interestingly, although AOA significantly improved embryonic development, we noted that normal fertilization rate in AOA group was lower. Subgroup analysis found that the lower normal fertilization rate in AOA group was only present in women aged ≥40 years. The explanation is that female’s age grew in AOA cycles compared to their own previous non-AOA cycles, and fertility rapidly declines over 40 years old [40]. Meanwhile, age-related reduction of oocytes retrieved partially contributed to the lower fertilization rate. Some studies reported that less retrieved oocytes are associated to total failed fertilization [41, 42]. However, progressing aging was inevitable in clinical practice when AOA was considered to be used to rescue previous poor embryo quality.

The study on sibling oocytes reported that AOA performed 1 hour after ICSI did not improve embryo and pregnancy outcomes in patients with previous poor embryo development but more than 70% normal fertilization rate [25]. They concluded that patients without fertilization problems might have normal Ca^2+^ signal pattern and normal development. Some studies have emphasized that AOA is not beneficial for all patients with oocyte activation deficiency [18, 26]. Besides, short of calcium during oocyte activation is not the only reason for poor embryo development. Sperm DNA damage, sperm chromatin abnormalities, oocyte abnormalities in structural proteins and mitochondria, or transcription factors and other regulatory proteins could also lead to poor prognosis of embryo [43, 44], which cannot be rescued by AOA. Maternal age and fertilization rate may act as significant reference indicators for application of AOA in cases with poor embryo development.

The lack of homogeneous AOA protocols impairs the inter-study comparison. We applied calcium ionophore at 15–30 minutes after ICSI in order to mimic natural calcium rise in conventional ICSI mode. Variations in Ca^2+^ response and oocyte activation rate between different AOA protocols have also been mentioned [34]. Double ionophore application were proposed to rescue some patients’ developmental problems, which might benefit from simulating a more physiologic calcium signal pattern [36]. Recombinant PLCζ protein can be considered for triggering Ca^2+^ oscillations in oocytes but there are still many limitations on its application, such as low activity, the use of fusion proteins, and unassessed post-implantation development [21]. Determining the scope of ionophore application and standardizing agents, dosage, timing, duration, and times of ionophore exposure are necessary [45].

Our results showed that calcimycin was a safe treatment in terms of pregnancy outcomes and congenital defect, confirming previous safety studies [35, 46]. Follow-up studies of children born following calcium ionophores activation reported reassuring results about neurodevelopmental and language developmental outcomes [47, 48]. But more large-scale prospective studies with long-term follow-up of AOA-born children are still needed.

The study included all day 3 and blastocyst transfers, fresh and frozen embryo transfers to observe the overall condition of clinical outcomes. More blastocysts were formed and frozen in AOA group, which contributed to more frozen embryo transfers. It has been reported that frozen or fresh transfer have no significant effects on clinical outcomes [49], while the effect of embryo stage when transferring is debatable [50–52]. It is commonly accepted that clinical pregnancy rate of blastocyst transfer is significantly higher than that of cleavage-stage embryo transfer [52–54]. There was part of included patients with relative normal day 3 good quality embryo rate in non-AOA cycles but significant lower good quality blastocyst formation rate whose pregnancy outcomes may be improved by early-stage embryo transfer and in vivo early embryo development [55]. Therefore, improved pregnancy outcomes by AOA were only observed in blastocyst transfer cycles in our study. In non-AOA group, embryos marginally developed into blastocyst stage and were transferred or frozen. AOA significantly improved the quality of blastocysts, allowing preferential selection of most optimal grade embryos for transfer that may have higher development potential [56]. In addition, AOA may improve post-implantation development that contributed to more satisfactory pregnancy outcomes. Admittedly, the present study is limited by small sample size and intrinsic deficits of retrospective analysis. We plan to study further with multicenter joint and expanded sample size in the future.

## Conclusion

In conclusion, calcimycin treatment is now mainly used for fertilization failure and its application for improving embryonic development is still non-routine. In this study, with a stratified analysis of age, we show for the first time that calcimycin can be applied as a feasible treatment in women aged < 35 years with poor embryo development in previous ICSI cycles.

## Supplementary Information

**Additional file 1.**

## Abbreviations

ART: assisted reproductive technology
IVF: in vitro fertilization
ICSI: intracytoplasmic sperm injection
PLCζ: phospholipase C zeta
Ca^2+^: Calcium
AOA: assisted oocyte activation
COH: controlled ovarian hyperstimulation
hCG: human chorionic gonadotropin
PN: pronuclear
BMI: body mass index
AMH: anti-Mullerian hormone.
